# Unlocking success: The power of change management in core facilities

**DOI:** 10.1038/s44319-025-00445-1

**Published:** 2025-04-23

**Authors:** Saskia Lippens, Katerina Hoskova, Ondrej Hradil, Jutta Steinkoetter, Henri G A M Van Luenen, Geert Van Minnebruggen, Danielle Hoyle

**Affiliations:** 1 VIB Technologies, VIB, Flanders Institute for Biotechnology, Ghent, Belgium; 2 CEITEC - Central European Institute of Technology, Masaryk University, Brno, Czech Republic; 3 Masaryk University, Brno, Czech Republic; 4 Max-Delbrück-Centrum für Molekulare Medizin (MDC), Berlin, Germany; 5 The Netherlands Cancer Institute, Amsterdam, The Netherlands; 6 Babraham Institute, Cambridge, UK

**Keywords:** Economics, Law & Politics, Methods & Resources, Science Policy & Publishing

## Abstract

Core facilities should be prepared to adapt to user demands and technological developments. Change Management strategies can help institution and facility management to efficiently communicate and implement necessary changes.

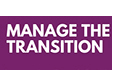

Core facilities are integral to research in the life sciences: as a centralised resource, they provide scientists with access to state-of-the-art technology and expertise that most individual labs cannot afford. Various terms are used to describe and label this form of centralized services, such as Science Facilities, Technology Units and Technology Platforms. For clarity, we use ‘core facility’ throughout this article.

More than just offering research services, core facilities enhance the efficiency and quality of research, they actively scout for new scientific and technological developments that could benefit research at their institution and they offer training for scientists to learn new methods and how to work efficiently with new instruments (Meder et al, 2016). On the institutional side, the centralization of services enables leadership to better manage their financial, capital and human resources economically and to collaborate more efficiently with other organisations by avoiding unnecessary duplication of services (Tranfield and Lippens, 2024).

It is therefore vital for a research organisation, be it a university or research institute, that core facilities remain relevant to the institution’s scientific mission and meet their researchers’ needs. Consequently, they need to constantly adapt to technological and scientific developments, changing economics, user demand and other factors. This could involve investing into new technologies or services, expanding services, resetting the scientific focus or even reducing or shutting down services. Here, we describe three case studies where core facilities had to adapt to external changes. In all cases, institutional and facility leadership used methods from so-called change management to successfully adapt their core facilities.

“It is vital for a research organisation, be it a university or research institute, that core facilities remain relevant to the institution’s scientific mission and meet their researchers’ needs.”

## Why change?

It is necessary for core facilities to regularly assess whether their activities still align with their initial purpose, whether they still meet scientists’ expectations and to be prepared for future technological advancements. This requires systematic evaluation incorporating feedback from multiple stakeholders and considering a wide range of performance indicators to identify a potential need for substantial changes.

Such change often means adopting new or emerging technologies either developed within a core facility, from an outside manufacturer or developed in collaboration with academic or commercial partners. Alternatively, an individual research group may successfully develop a technology or method to such an extent that it becomes necessary to manage this as a community resource and expand the scope of current research programmes. Acquisition of new assets may also be the result of change of an institution’s scientific direction, new PI recruitments or the changing focus of its scientists. Change may also be caused by personnel, operational or financial factors, including the loss of key users or facility staff. All these changes require organisations to adapt and to develop strategies that meet the current and future business needs in the most effective way.

Alongside adopting new capabilities, other changes include merging core facilities, forming new partnerships or outsourcing standardised services. Increasingly we are also seeing the development of cross-facility workflows or pipelines that necessitate a high degree of cooperation between cores. This includes multi-omics analyses, where the same set of samples are analysed across a series of core facilities to generate a wide range of different biological information. For example, single cells would be sorted by Flow Cytometry and then undergo multiple ‘omics’ characterisations to generate genomic, transcriptomic, proteomic and metabolomic data with the outputs being integrated by a Bioinformatics or Data Science team.

We therefore regard an appetite and a need for constant improvement as a positive attribute and core facility leaders and senior leadership teams should be proactive advocates for change. A proactive mindset means an organisation is better equipped and prepared for the future; waiting to react to factors that impose themselves increases the risk that the response is inappropriate.

“A proactive mindset means an organisation is better equipped and prepared for the future; waiting to react to factors that impose themselves increases the risk that the response is inappropriate.”

The ability to efficiently devise and implement change helps a core facility to maintain a competitive advantage and to deliver the best possible service for researchers. To support this exercise, the Core Facility Lifecycle recommendations, published by the EU-LIFE alliance, provides guidance and recommendations for navigating change and adaptation across all stages of core facility management (EU-LIFE, 2025). Whenever change is warranted, core facilities must be prepared to adjust their portfolio of services accordingly, necessitating the implementation of robust change management principles. We identified many commonalities across all stages of the life of a core facility, irrespective of the local governance arrangements of the individual organisations. This indicates that there is an opportunity to share best practises in core facility change management that would be broadly applicable within any environment.

## Navigating change: models and theories

Change is an inevitable consequence of working in a highly dynamic, research-intensive environment. However, it can be difficult to manage and implement; indeed it is estimated that more than 70% of change projects fail (Sturdy and Grey, 2003). Thus, it must be handled with great care and attention, taking a systematic approach to consider opportunity costs, limit any omissions and avoid moving too fast too soon.

In an attempt to overcome these inherent challenges, the change management discipline has been developing theories and models since the mid-20th century that provide support and guidance to organisations from idea to implementation. These models are wide-ranging in scope and nature, distinguished by factors such the frequency, scale and nature of the desired change. Whatever the structure of the model, they are all designed to make change as easy as possible, by identifying and overcoming resistance and supporting organisations in embedding the new norm.

Organisational change can elicit strong emotional reactions, which—depending on the drivers and intended outcomes—can have both positive and negative effects. Through our practise, we have identified that frequent and consistent communication is crucial, with senior management and ‘those in the know’ maintaining discretion until the strategy for change is fully developed. This limits the possibility of unintended consequences, including resistance, from uninformed stakeholders. We therefore recommend that managers use the rich library of resources developed by the change management field to support their decision-making processes in cases when core facilities must adapt in response to internal and external factors. Change management tools provide consistency and rationality, enabling stakeholders to systematically work through the changes within the wider context.

“Change management tools provide consistency and rationality, enabling stakeholders to systematically work through the changes within the wider context.”

**Figure 1:**
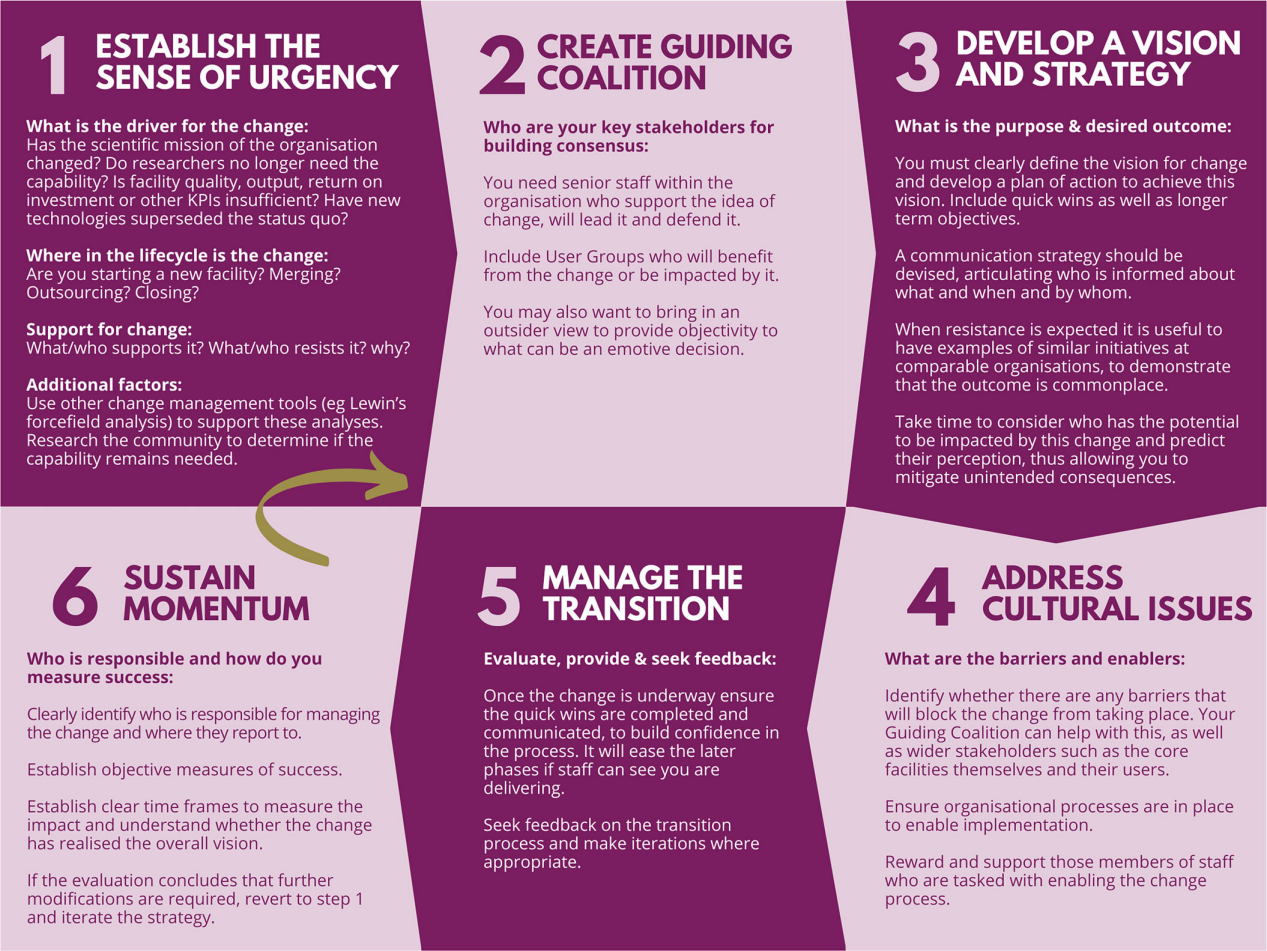
Stepwise model for supporting change within a core facility.

We are not prescribing colleagues to follow any one theory in particular; it is important to understand the context and apply an appropriate model. However, we have found that the N*-step models provide a practical framework for change as the stepwise approach offers a structure to consider, plan and enact the change initiative (Kanter et al, 1992; Kotter, 1996). These cyclical models encourage repetition or reconsideration of steps along the way to take into account new information, thereby providing a dynamic framework. Many authors have put forth ideas for models with varying steps; however, all models have some commonalities. Figure 1 shows a six-step model and articulates how each step could relate to change within a core facility environment (Open University, 2014). The 6th step “Sustain momentum” encourages monitoring and evaluation of the effectiveness of the change and recommends the option to further iterate when additional drivers of change emerge.

## Case studies of change management within core facilities

To provide support for a structured approach to managing change within the core facility lifecycle, we describe how we applied the cyclical step model to case studies from our organisations. These are not intended to be exhaustive commentaries of each case but examples of how leadership has followed the principles to the benefit of the organisation. In particular, we will discuss three cases at the Flanders Institute for Biotechnology (VIB) in Belgium, the Babraham Institute (BI) in Cambridge, UK, and the Central European Institute of Technology CEITEC in Brno, Czech Republic.

VIB found that service units were struggling to meet an increasing demand for bioinformatics analysis. Additionally, as bioinformatics skills were becoming more prevalent within individual research groups, it was decided to suspend centralised bioinformatics analysis as a distinct service. This was accompanied by a rise of data-intensive technologies beyond genomics, including proteomics, flow cytometry, structural biology and single-cell analysis. VIB took the decision to embed domain-specific bioinformaticians in their wet-lab core facilities. In parallel, a training unit was established to support both the scientific community and the sustainability of the embedded bioinformaticians and pipelines.

At the BI, it was becoming financially unsustainable to run the existing ‘workhorse’ DNA sequencer due to expensive consumables and poor economies of scale, creating uncompetitive prices for researchers. The institute therefore decided that outsourcing ‘routine’ sequencing to an academic partner, whilst retaining in-house bespoke capabilities for method development/R&D and diversification to new value-added activities was a more sustainable solution.

Despite previous investments in automation and instrumentation, use of the ‘stand-alone’ X-Ray Diffraction and Bio-SAXS Facility (CF X-Ray) at CEITEC was declining. To ensure the sustainability of the infrastructure, CEITEC determined that the facility should be merged with existing, complementary core facilities to benefit from expertise and economies of scale.

## Implementation of change

In all cases, it was internal factors that created pressure to change: changing volume and complexity of data alongside a democratisation of skills and expertise (VIB); financial pressures (BI); and a desire to see better returns on investment (CEITEC). All three institutions needed to retain existing capability but required an adjustment in how it was delivered to users. To better understand the drivers for change and what the outcome should look like, BI convened a User Group meeting to articulate the problem and solicit views about possible ways forward. This identified from the outset resisting forces such as concerns over turnaround times and flexibility versus the desire to reduce costs. Furthermore, they engaged with other members of the genomics community to understand how it is managed elsewhere and where outsourcing could take place. The separate cores at CEITEC were dependent on grant funding through Large Research Infrastructure projects, which included fixed term funding for their operations. The closure of these projects enabled the consolidation of these complementary activities.

The key stakeholders for change were similar across all three organisations: executive committees, heads of core facilities, heads of core programmes and user groups. Usually, the heads of programmes and the heads of core facilities supported and defended the changes, with the higher decision-making bodies eventually endorsing them. VIB was the only organisation to bring in an external advisory panel.

“The key stakeholders for change were similar across all three organisations: executive committees, heads of core facilities, heads of core programmes and user groups.”

At VIB the vision and strategy for change was developed by the head of programme and the head of the core facility, in consultation with human resources. Part of the plan was to determine where the work would be rerouted to, including the people who would be redeployed. The communication strategy required managers to first speak with those affected to clearly explain the vision and plan of action, and respond to concerns, before a communication was made to the wider community. The co-workers who were subject to the change trajectory initially voiced concerns that their skill set and expertise were not valued by upper management. In addition, the switch in direct line management also brought uneasiness for certain staff during the initial period. To address these concerns, it was important to give attention and time so that all parties involved could find comfort in the future setting.

The Babraham Institute undertook a series of financial and operational cost-benefit analyses to provide objective data. These identified that outsourcing would reduce direct costs to users but reduce income for a facility dependent on cost recovery; thus, it was imperative to diversify into other income-generating activities. The communication focused on the intention to retain an in-house Genomics Facility and the need for user input into how to make it sustainable. User Group meetings and surveys were used to develop these plans. The CEITEC strategy for change was to emphasise how users would benefit from the combined services portfolio and economies of scales offered by a single unit.

To address the cultural issues and help overcome barriers, VIB’s change managers predicted points of friction and identified team members to act as champions for their proposals. At BI and CEITEC, the main barriers were logistics, rather than linked to personnel. Consolidating data workflows and pipelines between core facilities within an organisation (CEITEC) and between organisations (BI) needed to be planned and implemented. Additionally, all the operational information—prices, websites, accounting, booking systems and so on—needed to be changed and updated. These were not barriers to the change itself but took considerable time and attention, because, if not managed well, would impede adoption by users.

## Outcomes

Upon implementation, all three organisations committed to evaluation and review of their change programmes. At VIB, the head of programme was supported by an external change consultant. Clear milestones were established at the planning stage and are being used to evaluate the benefits.

CEITEC performed an interim review 24 months after the facilities merged to evaluate the effect of the change and give recommendations to the head of the core facility on how to improve further. This identified that, to date, the merger has not brought the desired outcome, namely to attract more users, so it became necessary to increase specific activities to that end and closely monitor the cost-benefit analysis. This will include re-evaluating the objectives and iterating where necessary, keep the expert staff teams motivated, and broadening their knowledge and skills base. The facility will undergo further evaluation after 24 months.

After establishing outsourcing as a legitimate activity, the BI Genomics Facility retained some sequencing capability which allowed R&D or small-scale runs, enabling method development and offering bespoke workflows that would not be readily provided through their partners. The facility staff continued to observe the technology developments and needs of researchers. After almost four years of outsourcing, the facility has recently acquired new instrumentation allowing them to re-establish a cost-effective sequencing service. The driver for this change included reduced turnaround times and keeping finances ‘in-house’ to support financial sustainability. Additionally, the facility wanted to offer a bespoke and flexible project planning/provision, which the service provider was not able to readily provide. These changes followed the same decision-making processes as the original plan to outsource. The new instrument is now part of a diversified portfolio that includes QC, library preparation and short and long-read sequencing. This demonstrates the necessity of evaluation and how core facilities can and must be adaptable.

We hope these examples of how these institutions used the principles of established theories from change management to support their programmes provide useful context and support to colleagues who are facing similar challenges. We have created this resource, to be read in conjunction with our guidelines on core facility lifecycles (EU-LIFE, 2025), so colleagues across the core facility community can share in the expertise and knowledge we benefit from within our EU-LIFE working group and thus amplify the impact of that alliance.

“We hope these examples of how these institutions used the principles of established theories from change management provide useful context and support to colleagues who are facing similar challenges.”

## Supplementary information

Peer Review File
