# Supplementary material for: Unlocking success: The power of change management in core facilities
Source: EMBO Rep. 2025 Apr 23;26(9):2227–31. doi: 10.1038/s44319-025-00445-1 (PMC12069622; doi:10.1038/s44319-025-00445-1)
Supplement: Supplementary file 1 — Peer Review File [file 44319_2025_445_MOESM1_ESM.pdf]

EMBO reports

Unlocking Success

Saskia Lippens, Ondrej Hradil, Katerina Hoskova, Jutta Steinkoetter, Henri Van Luenen, Geert Van Minnebruggen, and Danielle Hoyle

Corresponding author(s): Danielle Hoyle (Danielle.Hoyle@babraham.ac.uk) , Saskia Lippens (Saskia.Lippens@vib.be)

Review Timeline:

Submission Date:	6th Mar 25
Accepted:	31st Mar 25

Editor: Holger Breithaupt

Transaction Report: This article was editorially reviewed.
